# Supplementary material for: Safe and Effective Cynomolgus Monkey GLP—Tox Study with Repetitive Intrathecal Application of a TGFBR2 Targeting LNA-Gapmer Antisense Oligonucleotide as Treatment Candidate for Neurodegenerative Disorders
Source: Pharmaceutics. 2022 Jan 15;14(1):200. doi: 10.3390/pharmaceutics14010200 (PMC8780845; doi:10.3390/pharmaceutics14010200)
Supplement: Supplementary file 1 [file pharmaceutics-14-00200-s001.zip › pharmaceutics-1480672-supplementary.pdf]

pharmaceutics

Supplementary Materials: Safe and Effective Cynomolgus Monkey GLP—Tox Study with Repetitive Intrathecal Application of a TGFBR2 Targeting LNA-Gapmer Antisense Oligonucleotide as Treatment Candidate for Neurodegenerative Disorders

Sebastian Peters, Eva Wirkert, Sabrina Kuespert, Rosmarie Heydn, Siw Johannesen , Anita Friedrich, Susanne Mailänder, Sven Korte, Lars Mecklenburg, Ludwig Aigner, Tim-Henrik Bruun and Ulrich Bogdahn

Supplementary information

Table S1. Timepoints of plasma sampling.

Overview of the different time points for plasma collection.

Group/Sex	Time Points	Sample Time (Hours Postdose)								
		0	1	2	4	8	12	24	48	168
1 M/F	Days 1 and 85	X	X	X	X	X	X	X	X	X
2 M/F	Days 1 and 85	X	X	X	X	X	X	X	X	X
3 M/F	Days 1 and 85	X	X	X	X	X	X	X	X	X
4 M/F	Days 1 and 85	X	X	X	X	X	X	X	X	X

F = Females; M = Males.

Group/Sex	Time Points	Sample Time (Before Dosing)
1 M/F	Days 15, 29, and 57	X
2 M/F	Days 15, 29, and 57	X
3 M/F	Days 15, 29, and 57	X
4 M/F	Days 15, 29, and 57	X

F = Females; M = Males.

Group/Sex	Time Points	Sample Time
1 M/F	Days 98, 105, 112, 119, 133, 147, 175, and 182	X
2 M/F	Days 98, 105, 112, 119, 133, 147, 175, and 182	X
3 M/F	Days 98, 105, 112, 119, 133, 147, 175, and 182	X
4 M/F	Days 98, 105, 112, 119, 133, 147, 175, and 182	X

F = Females; M = Males.

Table S2. Timepoints of CSF sampling.

Overview of the different time points for CSF collection.

Group/Sex	Time Points	Sample Time (Hours Postdose) 0	8	24
1 M/F	Days 1, 15, 29, 57, and 85	X	X	X
2 M/F	Days 1, 15, 29, 57, and 85	X	X	X
3 M/F	Days 1, 15, 29, 57, and 85	X	X	X
4 M/F	Days 1, 15, 29, 57, and 85	X	X	X
1 to 4 M/F	Days 43 and 71 (±1 day)	X	X	X
1 to 4 M/F[a]	Day 92			Before Necropsy

F = Females; M = Males.

a Only for terminal sacrifice animals.

Group/Sex	Time Points	Sample Time
1 M/F	Days 98, 105, 112, 119, 133, 147, 175, and 182	X
2 M/F	Days 98, 105, 112, 119, 133, 147, 175, and 182	X
3 M/F	Days 98, 105, 112, 119, 133, 147, 175, and 182	X
4 M/F	Days 98, 105, 112, 119, 133, 147, 175, and 182	X

F = Female; M = Male.

Table S3. Neurobehavioral Observations.

Overview of the relevant parameters for neurobehavioral observations

Parameters	Explanation	Score	Range
Part 1			
Checking Vocalization	Mouth partially closed, with abdominal contractions	-1 to +1	0 = normal
Communicative Vocalization	Mouth open, often with increasing volume	-1 to +1	0 = normal
Grooming	Auto-grooming behavior	-1 to 0	0 = normal
Posture	Head and/or body posture	-2 to 0	0 = normal
Balance/Coordination	Limb coordination and/or postural balance	-4 to 0	0 = normal
Locomotor Activity	Activity level within the home cage	-1 to +1	0 = normal
Tremors	Involuntary muscle contractions that cause oscillatory movement of the body, head, or limbs	0 to +2	0 = absent
Twitches/Jerks	Involuntary muscle contractions that cause the animal to abruptly jerk or twitch	0 to +1	0 = absent
Muscle Cramping	Involuntary muscle contractions that persist for seconds to minutes	0 to +1	0 = absent

Parameters	Explanation	Score	Range
Part 1			
Stereotypy	Repetitive, apparently purposeless or ritualistic postures or movements (e.g., circling behavior or retropulsion)	0 to +2	0 = absent
Alertness	Noticeable reaction to a technician approaching the cage	-3 to +1	0 = normal
Offensive Aggression	In response to a technician approaching the cage (moves to the front of the cage, bears teeth, and possibly vocalizes)	0 to +2	0 = normal
Defensive Aggression	In response to a technician approaching the cage (moves to the back of the cage, bears teeth, and possibly vocalizes)	0 to +2	0 = normal
Reaction to Visual Stimuli	Tracking of an object's movement in front of the cage	-1 to +1	0 = normal
Reaction to Acoustic Stimuli	Response to an acoustic stimulus from a hidden source	-1 to +1	0 = normal
Part 2			
Muscle Tone	Assessed by palpation of limbs and the abdomen	-2 to +2	0 = normal
Grip Strength	Qualitatively assessed during removal of the animal from the cage or during further handling (e.g., ability to grip a part of the cage)	-3 to 0	0 = normal
Lacrimation	By visual inspection	0 to +2	0 = absent
Salivation	By visual inspection	0 to +2	0 = absent
Part 2 - Measurement			
Body Temperature	Rectal measurement (°C)		

Table S4. Summary of parameters tested within plasma, CSF, and urine.

Overview of the tested parameters for hematology, clinical chemistry, CSF parameters, and urinalysis.

Hematology Tests

Reticulocyte Count (RETI.)	Lymphocytes (LYM.)
Absolute Reticulocyte Count (TRET.)	Monocytes (MONO.)
Red Blood Cell Count (RBC.)	Eosinophils (EOS.)
Hemoglobin (HGB.)	Basophils (BASO.)
Hematocrit (HCT.)	Absolute Neutrophils (ANEU.)
Mean Corpuscular Volume (MCV.)	Absolute Lymphocytes (ALYM.)
Mean Corpuscular Hemoglobin (MCH.)	Absolute Monocytes (AMON.)

Mean Corpuscular Hemoglobin Concentration (MCHC.)
White Blood Cell Count (WBC.)
Neutrophils (NEUT.)
Absolute Eosinophils (AEOS.)
Absolute Basophils (ABAS.)
Platelet Count (PLT).

Clinical Chemistry Tests

Total Bilirubin (TBIL.)
Creatinine (CREA.)
Blood Urea (BU.)
Glutamate Dehydrogenase (GLDH.)
Aspartate Aminotransferase (AST.)
Alanine Aminotransferase (ALT.)
Alkaline Phosphatase (ALP.)
Gamma Glutamyl Transferase (GGT.)
Glucose (GLU.)
Total Cholesterol (CHOL.)
Triglycerides (TRIG.)
Inorganic Phosphorus (PHOS.)
Calcium (CA.)
Sodium (NA.)
Potassium (K.)
Chloride (CL.)
Total Protein (TP.)
Albumin (ALB.)
Globulin (GLOB.)
Albumin/Globulin Ratio (A:G.)

CSF Chemistry Tests

CSF Glucose (CGLU.)[a]
CSF Inorganic phosphorus (CPHO.)[a]
CSF Calcium (CCA.)[a]
CSF Sodium (CNA.)[a]
CSF Qualitative WBC (CQWBC.)
CSF Potassium (CSFK.)[a]
CSF Chloride (CCL.)[a]
CSF Total protein (CTP.)[a]
CSF Albumin (CMIA.)[a]
CSF Qualitative RBC (CQRBC.)

Urinalysis Tests

Urine Volume (UVOL.)
Urine Specific Gravity (USGR.)
Urine pH (UPH.)
Urine Leukocytes (ULEU.)[a]
Urine Nitrite (UNIT.)[a]
Urine Protein (UPRO.)[a]
Urine Glucose (URGL.)[a]
Urine Ketones (UKET.)[a]
Urine Urobilinogen (UUBG.)[a]
Urine Bilirubin (UBIL.)[a]
Urine Occult Blood (UOBL.)[a]
Urine Color (UCOL.)
Urine Clarity (UCLA.)
